# Miracle fruit seed as a potential supplement for the treatment of learning and memory disorders in Alzheimer’s disease

**DOI:** 10.3389/fphar.2022.1080753

**Published:** 2023-01-11

**Authors:** Xue-Yan Huang, Lu-Lu Xue, Ting-Bao Chen, Li-Ren Huangfu, Ting-Hua Wang, Liu-Lin Xiong, Chang-Yin Yu

**Affiliations:** ^1^ Department of Neurology, Affiliated Hospital of Zunyi Medical University, Zunyi, Guizhou, China; ^2^ State Key Laboratory of Biotherapy, Sichuan University, Chengdu, Sichuan, China; ^3^ Laboratory Animal Department, Kunming Medical University, Kunming, Yunnan, China; ^4^ Department of Anesthesiology, Affiliated Hospital of Zunyi Medical University, Zunyi, Guizhou, China

**Keywords:** Alzheimer’s disease, miracle fruit seeds, molecular docking, Alzheimer’s disease pathway, insulin signaling pathway, AKT1, EGFR

## Abstract

Currently, the treatment of Alzheimer’s disease (AD) is still at the stage of symptomatic treatment due to lack of effective drugs. The research on miracle fruit seeds (MFSs) has focused on lipid-lowering and antidiabetic effects, but no therapeutic effects have been reported in AD. The purpose of this study was to provide data resources and a potential drug for treatment of AD. An AD mouse model was established and treated with MFSs for 1 month. The Morris water maze test was used to assess learning memory function in mice. Nissl staining was used to demonstrate histopathological changes. MFSs were found to have therapeutic implications in the AD mouse model, as evidenced by improved learning memory function and an increase in surviving neurons. To explore the mechanism of MFSs in treating AD, network pharmacological approaches, Gene Ontology (GO), Kyoto Encyclopedia of Genes and Genomes (KEGG), and molecular docking studies were carried out. Based on the network pharmacology strategy, 74 components from MFS corresponded to 293 targets related to the AD pathology. Among these targets, AKT1, MAPK3, ESR1, PPARG, PTGS2, EGFR, PPARA, CNR1, ABCB1, and MAPT were identified as the core targets. According to the relevant number of core targets, cis-8-octadecenoic acid, cis-10-octadecenoic acid, 2-dodecenal, and tetradecane are likely to be highly correlated with MFS for AD. Enrichment analysis indicated the common targets mainly enriched in AD and the neurodegeneration-multiple disease signaling pathway. The molecular docking predictions showed that MFSs were stably bound to core targets, specifically AKT1, EGFR, ESR1, PPARA, and PPARG. MFSs may play a therapeutic role in AD by affecting the insulin signaling pathway and the Wnt pathway. The findings of this study provide potential possibilities and drug candidates for the treatment of AD.

## Introduction

AD is a neurodegenerative disease, the most common symptoms of which are memory impairment and cognitive decline, and is the most common contributor to dementia. The drugs currently approved by Food and Drug Administration for therapeutic use are primarily for the treatment of AD symptoms, including acetylcholinesterase inhibitors [donepezil (Knapp, et al., 2017), galantamine (Baakman, et al., 2022), and rivastigmine (Jia, et al., 2019)] and N-methyl-D-aspartate antagonist [memantine (Grossberg, et al., 2018)]. However, relevant clinical studies also suggest that the benefit of donepezil in use is not evident (Birks and Harvey, 2018). Galantamine is less effective in improving memory and executive functioning disorders, with significant gastrointestinal side effects (Leijenaar, et al., 2020). Memantine is also used in combination therapy for AD, with no significant symptom improvement and increased economic costs (Knapp et al., 2017). The research for new treatments for AD has, therefore, become a pressing issue.

In recent years, the recognition of AD has been constantly updated, and medicinal plants are recognized for their synergistic effects of multiple chemical components, multiple pathways and multi-target mechanisms, and their advantages in the treatment of chronic, polygenic, and complex diseases (Perry, et al., 1999; Rao, et al., 2012; Dey, et al., 2017; Gregory, et al., 2021). People are interested in treating AD plants because they have fewer side effects compared to synthetic drugs. Also, because of the clinical trial failures and multiple sides of synthetic drugs, the development of phytotherapy has received close attention from the public and the scientific community (Vegh, et al., 2019). Many foods and nutritional drugs are claimed to have memory-improving effects, including *Ginkgo biloba* (Singh, et al., 2019; Nowak, et al., 2021), dihydromyricetin (Martínez-Coria, et al., 2019), linalool (Hosseini et al., 2021), fig leaves (Sohn et al., 2021), *Schisandra* polysaccharides (Sun et al., 2014), *Nardostachys jatamansi* (Anupama et al., 2022), tea (Baranowska-Wójcik, et al., 2020), *Forsythia* (Yan et al., 2017), curcumin (Mithu et al., 2014), and acidic polysaccharose (Mithu et al., 2014).

*Synsepalum dulcificum*, also known as the miracle fruit, belongs to the mangosteen family. This plant is local to the forested areas of tropical West Africa and can be found growing wild alongside the Gulf of Guinea (Tchokponhoué, et al., 2019; Tchokponhoué, et al., 2021). It is so named because its fruit can turn sour into sweet when eaten with other acidic foods such as lemons, capers, and kimchi. This is related to the fact that miraculin protein, a specific component of the miracle fruit, activates the human sweet taste receptor T1R2–T1R3 in a dependent manner (Sanematsu, et al., 2016). This well-known function is now also used in the taste function of cancer chemotherapy patients (Wilken and Satiroff, 2012). Traditionally, it is used in West Africa mainly for the treatment of diarrhea and cough. After the 1960s, the mystery fruit was introduced to subtropical and tropical areas of China, including Hainan, Yunnan, Guangdong, Guangxi, and Fujian provinces (Cheng, 2000). The miracle fruit is currently found to be of use and economic significance in a variety of industries and has been approved for safety in the European Union as a food supplement (excluding pregnant and lactating women adults) (Turck, et al., 2021). The safety of miracle fruits was assessed by measuring their lectin levels, and it was found that miracle fruits can be taken orally without cooking like fruits such as blueberries (Menéndez-Rey, et al., 2021). This characteristic has brought more attention to the edible and medicinal value of the mystery fruit.

Current research on the miracle fruit has focused on the pulp and leaves, with less research on the seeds. The medical value of pulp is mainly focused on diarrhea (Offiah, et al., 2011), antidiabetic effects (Haddad, et al., 2020), anti-oxidation effects, etc. The leaf extract has the function such as presence of anti-hyperuric acid (Shi, et al., 2016; Chen, et al., 2018), inhibition of oxidative damage, and anti-mutagenicity (Chen, et al., 2015). Research on seeds has focused on cholesterol-lowering (Huang, et al., 2020) and antidiabetic effects (Han, et al., 2019). MFS oil has also been reported to treat breakage and damaged hair in women because of the phytochemicals and nutrients it contains (Del Campo, et al., 2017). In addition, studies have found that wristbands containing MFS oil can affect musculoskeletal performance and improve motor skills in the hands and fingers of healthy adults (Gorin, et al., 2018). MFS is mainly composed of the following components: ash, crude fiber, crude fat, reducing sugars, polyphenols, polysaccharides, fatty acids, amino acids, and mineral elements. Comparing these seeds to the common medicinal seeds (including *Perilla* seeds, palm seeds, and trillium seeds), the ash, crude protein, and polysaccharides of the MFS are higher than those of other plants, and they contain a variety of amino acids and mineral elements required by the human body, making them highly valuable for food and medicinal purposes. The seeds are high in amino acids and phyto-polyphenols compared to other seeds, with about 9.02 g/100 g of seeds in amino acids and 11.56 mg/g of seeds in phyto-polyphenols. Of the amino acid composition, 40.69% of total essential amino acids, 19.95% of total branched-chain amino acids, and a high content of essential and potent amino acids (63.75%) were present. The minerals of the mystery fruit seeds were measured and found to be typically high in potassium and low in sodium (Ma, et al., 2016). Such properties are beneficial in improving the potassium–sodium balance in the body and in the prevention and treatment of cardiovascular diseases and diabetes (Mohammadifard, et al., 2019; Zanetti, et al., 2020).

Related studies have shown that although the antioxidant activity in the seeds was lower than that of the miracle fruit peel and pulp, the seeds contributed 49.45% of the free antioxidant activity, 76.41% of the bound antioxidant activity, and 58.56% of the total antioxidant activity as they comprised about 66% of the total solids ((Inglett and Chen, 2011). Compared to the pulp and leaves, the seeds have better storability and transportability. Research on MFS is currently focused on the cholesterol-lowering and blood sugar-lowering components. The mechanism of lowering cholesterol is mainly thought to be related to the richness of triterpenoids. The lowering of blood glucose level is thought to be associated with its stimulation, promoting the expression of PI3K and GLUT4 and activating the insulin pathway in type 2 diabetic patients (Han, et al., 2019; Huang, et al., 2020). Therefore, MFS is a good source of antioxidant food. Diabetes is currently thought to be associated with AD through the insulin resistance pathway (Burillo, et al., 2021; Idowu et al., 2022). It is well-known that insulin can regulate GLUT4, which has an important role in brain glucose metabolism. A decrease in GLUT4 has also been found in the brains of postmortem AD patients (de la Monte and Wands, 2005). According to reports, insulin can mediate the growth, metabolism, and survival of neurons and glia cells and affects synaptic function (Talbot, et al., 2012). In AD patients, a reduced rate of glucose metabolism was found, especially in brain regions associated with memory (Mosconi, et al., 2008; Mistur, et al., 2009; Chen and Zhong, 2013; Croteau, et al., 2018), and abnormalities in glucose metabolism may precede the decline in cognitive function (Chen, et al., 2010). In addition, abnormal cholesterol metabolism is now considered as a potential risk factor for AD and is expected to be a new target for AD treatment (Loera-Valencia, et al., 2019; van der Kant, et al., 2019; Sáiz-Vazquez, et al., 2020). Notably, transcriptomic analysis also revealed impaired cholesterol biosynthesis in brain regions susceptible to AD pathology (Varma, et al., 2021). Brain cholesterol levels are closely related to the function of neurons, glial cells, and memory formation (Li, et al., 2022).

MFSs showed therapeutic implications for insulin resistance and cholesterol metabolism. This led us to speculate that MFS has therapeutic implications for AD and to implement the reasons for this study. This study investigates the effects of the methanolic extract of MFSs using an AD transgenic mouse model and explored the intrinsic mechanisms.

## Material and methods

### Animals

We used C57BL/6JOlaHsd150 inbred mice aged 9 months (Experimental Animal Center of Kunming Medical University), weighing 25 g–30 g at the beginning of the experiment. Transgenic mice with familial two AD mutations (2 × FAD) were purchased from the Jackson laboratory, 9-month-old males. Also, 2 × FAD overexpresses the human APP SWE and PS1DEL9 genes, age-related neuropathology overexpression of Aβ and age-dependent cognitive, and learning dysfunction. The 2 × FAD mice were transgenic hemizygotes, and non-transgenic wild-type (WT) littermates were used as controls. Genotyping was carried out by quantitative real-time polymerase chain reaction (qRT-PCR) analysis of tail DNA. All mice were housed, 5–6 mice/cage, under standard laboratory conditions (temperature: 22°C ± 1°C, humidity: 60%). Food and water were freely available under a 12-h dark–light cycle in standard cages. All manipulations were carried out during the light cycle.

### Miracle fruit seed methanol extract material and preparation

The methanol extract of MFS was obtained from the School of Pharmacy, Zunyi Medical University. The seeds were air-dried and then crushed into powder. A portion of the pulverized sample (408.4 g) was extracted in methanol (2.042 L) by maceration for 72 h. After complete infusion, the mixture was filtered, and the filtrate was concentrated under reduced pressure in a rotary evaporator and freeze-dried. The extract powder was obtained and stored away from light for further use.

### Grouping of animals

**FIGURE 1:**
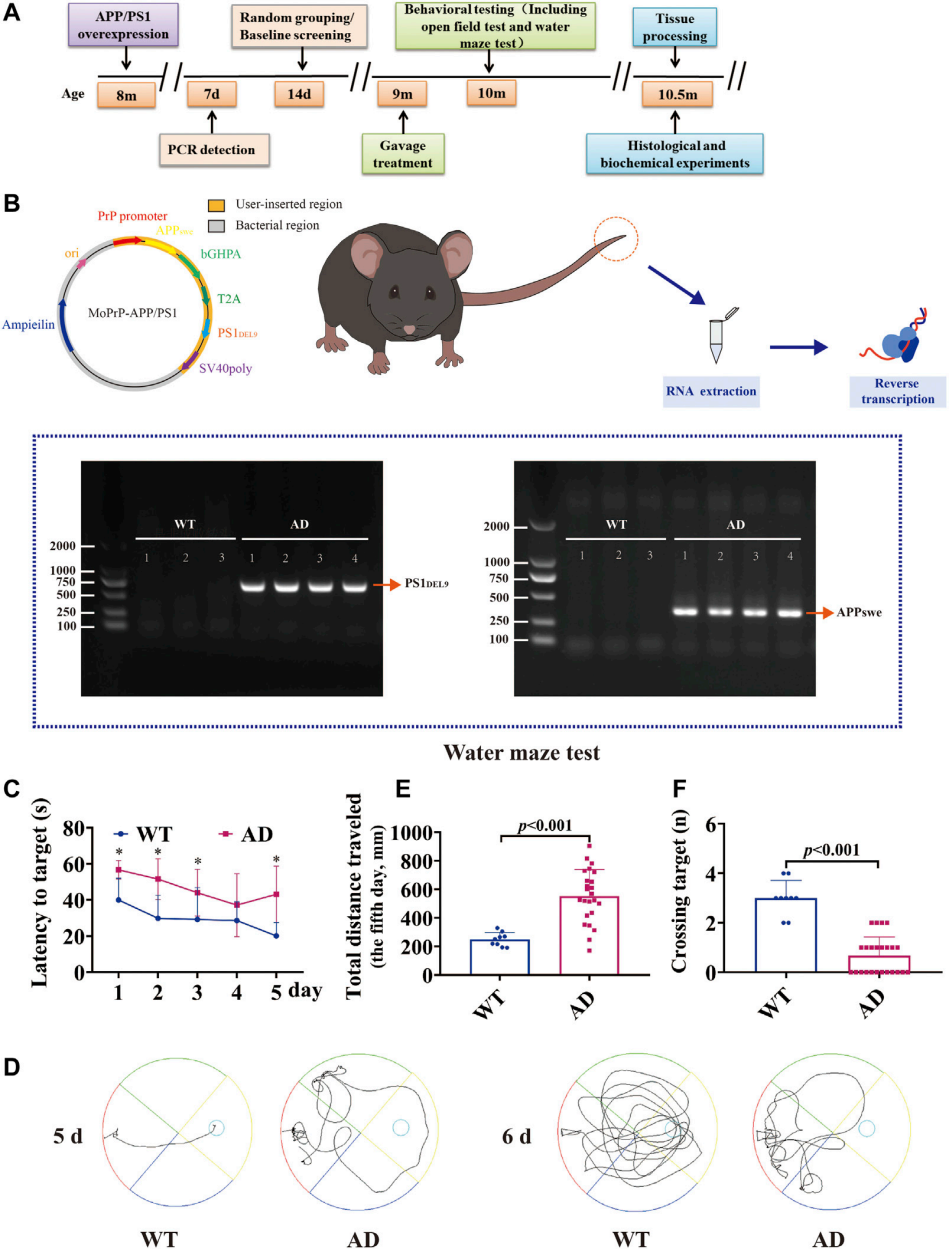
Experimental program and construction of AD transgenic mice. Mice were genotyped for tail detection and behavioral testing at 8 months of age, and gavage treatment was started at 9 months of age. Behavioral tests (including the open-field test and water maze test) were performed at 10 months of age (1 month of gavage treatment), and mice were executed for biochemical analysis the day after behavioral tests were completed. The expression of APP/PS1 in the tail of mice was identified using PCR. A bright band was visible at 350 bp and 608 bp compared to WT. APPswe/PS1DEL9 double transgenic AD mice were successfully constructed. The Morris water maze test suggested that 9-month-old AD model mice (APP/PS1 overexpression) already showed significant learning memory dysfunction compared to wild-type mice of the same age (data are presented as mean ± standard deviation) (WT, *n* = 9; AD, *n* = 24). **(A)** Experimental program. **(B)** Vector construction of APPswe and PS1DEL9 homologous recombination sequences and PCR detection of mouse genes. **(C)** Escape latency (seconds) during platform trials (* indicates WT compared to the AD model group; *p* < .05). **(D)** Trajectory diagram of the last day of training and day 6 of testing for the Morris water maze test. **(E)** Distance traveled (centimeters) on the last day of training in the Morris water maze test. **(F)** Number of crossings over the target platform of the Morris water maze test at day 6.

All animals were 9-month-old male mice with four groups (Table 1), namely, the WT group, AD control group, and AD-2 mg/kg (dose of MFS) and AD-6 mg/kg groups (dose of MFS). Before conducting this study, we used error degrees of freedom to estimate the number of mice needed (Dell, et al., 2002). Finally, we chose eight mice per group as the experimental number. The mice completed the rat tail PCR assay and Morris water maze baseline screening and were treated with once-daily gavage starting at 9 months of age, with the gavage dose calculated based on body weight (.1 mL (mL) gavage dose/10 g mouse body weight). The experimental process is shown in Figure 1A. The methanol extract mixture of MFS was dissolved in double-distilled water (drug concentrations were .2 mg/mL and .6 mg/mL, respectively), mixed with ultrasound until completely dissolved, stored in a 4°C refrigerator, and taken out when used. The 2 × FAD mice were treated with 2 mg/kg (group 3) or 6 mg/kg (group 4), depending on body weight by gavage once daily for 3 months, starting at 9 months of age.

**TABLE 1:** Experimental groups of mice.

Group	Abbreviation	Description
1	WT	Wild-type mouse not treated
2	Control	AD model mouse treated with distilled water
3	2 mg/kg	AD model mouse treated with 2 mg/kg methanol extract of miracle fruit seed
4	6 mg/kg	AD model mouse treated with 6 mg/kg methanol extract of miracle fruit seed

All animal experiments conformed to the Guide for the Care and Use of Laboratory Animals, published by the National Institutes of Health. The animal surgery was legally approved and performed by the Animal Welfare Ethics Committee of Zunyi Medical University (approval number: ZMU21-2203-615). Animal stress and the use of animals were minimized.

### Behavioral tests

#### Morris water maze

The Morris water maze was performed to evaluate the spatial learning and memory capacity of the mice at nine and 10 months of age after gavage. The pool is divided evenly into four quadrants, with the platform located in the center of one of the quadrants. Edible white veggies were added to the pool with the aim of hiding the small round table in the pool water. Each mouse was trained in four quadrants for 60 s (s) per day for five consecutive days. During the training process, the mice failed to find the hidden platform within 60 s, and the experimenter gave instructions. On the 6th day of the experiment, the platform was removed, and the 60-s exploration training began. The mice were placed in the water from the other side of the original platform quadrant, and the time spent in the target quadrant (the quadrant where the platform was originally placed) was recorded. The time the mouse took to enter the quadrant and the number of times the mouse passed the platform were used as measures of spatial memory. Finally, at the end of the experiment, the mice were air-dried and placed in their home cage. Training intervals were 15–20 min.

#### Open-field test

The mice were tested at nine and 10 months in an open-field experiment. The open-field test was chosen to further assess the movement (motor function), autonomous behavior, exploratory behavior, and tension of the experimental animals in their new environment. The mice were placed in a room, a 30 cm × 30 cm × 35 cm open area with appropriate lighting conditions and a video camera fixed on top. Each mouse was placed in the middle of the open area and allowed to explore freely for 10 min. The total distance and average speed over a 10-min period were then automatically recorded by Supermaze, a video tracking software system supplied by NewSoft Information Technology (Shanghai, China), as an indicator of exercise activity. After each trial, the walls and floors of the open field were cleaned.

#### Tissue harvest

The mice were anesthetized with 2.5% isoflurane and then euthanized. The eyes were removed, and the whole blood of the mice was taken out. Their chest was opened, and the tip of the perfusion tube was fixed to the ascending aorta. Then, 20 mL–30 mL of normal saline (.9% sodium chloride) was injected at a constant rate to clean the blood, and brain tissue was exposed and collected. The right brain was fixed in 4% paraformaldehyde solution for 5 days, embedded using paraffin, and sectioned for morphological examination. The left brain was harvested and frozen to −80°C at room temperature for later molecular examination.

#### Nissl staining

Neuronal cells in the cortical and hippocampal sections were observed by Nissl staining. The cut brain slices (4 µm) were baked for 1 h in a 60°C oven. The brain slices were dewaxed and placed in a Nissl staining solution (Beyotime, C0117) to react for 15 min. Then, they were washed in distilled water, dehydrated in graded concentrations of ethanol (70%, 80%, 90%, and 100%), cleared in xylene, and finally capped with neutral balsam to seal the sections. Whole-section scanning light microscopy was performed for dark and surviving neurons (digital whole section scanner, Pannoramic MIDI, magnification × 200, × 400). Five random fields (× 200) were selected by blind observers and used to quantify the number of positive cells. Three sections were selected for each animal, and the mean of the cell counts in the right hippocampus and cortex [including cortical, hippocampal cornu ammonis 1(CA1), CA2, CA3, and dentate gyrus (DG) areas] was provided for each animal.

### Quantitative real-time PCR

The prepared cortex and hippocampus were homogenized and lysed. Total RNA was extracted with TRIzol reagent (Takara BioInc., Otsu, Japan) and then reversely transcribed into cDNA with Revert Aid™ First Strand cDNA Synthesis Kit (Thermo, United States) and All-in-One miRNA First Strand cDNA Synthesis Kit (GeneCopoeia). The qRT-PCR was then performed to detect the relative expression of mRNA, and APP and PS1 were detected at nine months of age. The primer sequences are shown in Table 2. Next, the reaction was performed in a DNA thermal cycler (ABI 7300), according to the following standard protocol: one cycle of 95°C for 5 min, 40 cycles of 95°C for 10 s, annealing of 52°C for 20 s, and extension of 72°C for 20 s. Relative expressions were calculated with normalization to GAPDH values by using the 2^−ΔΔCT^−11^−ΔΔCT^ method.

**TABLE 2:** Primer information.

	Forward	Reverse
APP	5′GAC​TGA​CCA​CTC​GAC​CAG​GTT​CTG 3′	5′CTT​GTA​AGT​TGG​ATT​CTC​ATA​TCC​G 3′
PS1	5′AAT​AGA​GAA​CGG​CAG​GAG​CA 3′	5′GCC​ATG​AGG​GCA​CTA​ATC​AT 3′
GAPDH	5′AGG​TCG​GTG​TGA​ACG​GAT​TTG 3′	5′GGG​GTC​GTT​GAT​GGC​AAC​A 3′

### Collection of the targets of miracle fruit seeds and correlation with Alzheimer’s disease pathology

The main components in MFSs were obtained from the literature. To collect target information for the compounds in the MFS, the chemical components already present in the traditional Chinese medicine systems pharmacology database and analysis platform (TCMSP) database were screened for active ingredients using oral bioavailability (OB ≥ 30%) and drug-likeness (DL ≥ .18) values. The remaining components for which detailed information was not available in the TCMSP database were predicted using SwissTargetPrediction (http://www.swisstargetprediction) (Daina, et al., 2019). SDF and 2-dimensional (2D) chemical structures were obtained from the PubChem database (https://pubchem.ncbi.nlm.nih.gov/) (Kim, et al., 2016), which is the largest free database of organic small-molecule bioactivities. Particularly, 2D structures of components were input into SwissTargetPrediction, and the target species was set as *Homo sapiens*. Subsequently, the target information of MFSs was collected and organized. Additionally, the targets of AD were obtained from the GeneCards database (https://www.genecards.org/) (Fishilevich, et al., 2017). Afterward, we obtained the intersection of AD-related genes and the potential targets of MFSs. The common targets were the MFS targets involved in the AD pathway. In the end, the UniProt protein database (https://www.uniprot.org/) (UniProt, 2021) was used to standardize and integrate protein targets and genes.

### Protein–protein interaction network construction and screening of its core targets

The PPI network of the target protein was collected with the STRING database (https://cn.string-db.org/) (von Mering, et al., 2005), which was used to establish connections between AD and MFS-related intersection targets. The analysis results were saved as TSV files and imported into Cytoscape software (http://www.cytoscape.org/, ver. 3.6.0) (Shannon, et al., 2003) for visualization. The organism was set as *Homo sapiens*. In the PPI network, betweenness centrality (BC) refers to the degree of mutual independence between nodes. The higher the BC, the more control a node will have over the network because more information will pass through that node, and the more important that node will be in the PPI network. The computation of BC is calculated by network analysis (a plug-in for Cytoscape) and the top 10 targets ranked by BC were selected as the core targets.

### GO and KEGG enrichment analyses

The Metascape database (https://metascape.org/gp/index.html#/main/step1) (Zhou, et al., 2019) was used to carry out the GO and KEGG pathway analyses of those intersection targets. GO enrichment analysis included molecular function (MF), biological process (BP), and cellular composition (CC). GO enrichment analysis and top 20 KEGG pathways sorted by the *p*-value were visualized using an online tool (http://www.bioinformatics.com.cn/).

### Molecular docking

To verify the binding of the core target to its corresponding MFS components, the 3D molecular structure of the MFS was downloaded from the PubChem database. The high-resolution crystal structures of the target protein were obtained from the RCSB Protein Data Bank (PDB database, http://www.rcsb.org/) (Burley, et al., 2017). The binding affinity between MFS compounds and AD targets was performed with AutoDock 4.2 and AutoDockTools (ADT). Finally, the interaction ability between the protein and the ligand was assessed by the scoring of the binding energy, and visualized and embellished by using PyMOL software.

### Statistical analysis

Statistical analysis was performed using SPSS 21.0 statistical software (IBM) and graphs were generated using Prism 6 (GraphPad). The data were first tested for normality. With data conforming to normal distribution, for comparisons between three or more groups, one-way analysis of variance (ANOVA) and Tukey’s *post-hoc* analysis were applied. Two groups of data were analyzed by *t*-test. Data that did not conform to the normal distribution were analyzed using the Kruskal–Wallis test. Data are presented as mean ± standard deviation (x ± SD). When *p* < .05, the difference was statistically significant.

## Results

### Construction and characterization of AD transgenic mice

The 2 × FAD transgenic mice purchased from the Kunming Medical University Laboratory Animal Department, overexpressing human APP and PS1 transgenes. To verify whether APP/PS1 was overexpressed, the expression of APP/PS1 in mice was detected by PCR, and non-transgenic wild-type littermates were used as controls (Figure 1B). Specific primers were synthesized for the human APP and PS1 genes, with amplification fragments of 350 bp for APP and 644 bp for PS1: 1) APP amplification primers: sense 5′-GAC​TGA​CCA​CTC​GAC​CAG​GTT​CTG-3′, anti-sense 5′-CTT​GTA​AGT​TGG​ATT​CTC​ATA​TCC​G-3'; 2) PS1 amplification primers: sense 5′-AAT​AGA​GAA​CGG​CAG​GAG​CA-3′, anti-sense 5′-GCC​ATG​AGG​GCA​CTA​ATC​AT-3'. Reaction conditions: one cycle of 95°C for 5 min, 40 cycles of 95°C for 30 s, annealing of 60°C for 30 s, and extension of 72°C for 10 min. The data showed a bright band at 350 bp (APP) and 608 bp (PS1) compared with WT, which was consistent with the expected position of the target gene fragment (Figure 1B).

#### Baseline screening

Nine-month-old WT male C57BL/6J mice and 2 × FAD mice were screened at baseline using the Morris water maze test to assess the learning and memory function. The results showed that compared with the WT group, the AD model mice had evident spatial learning and memory dysfunction in the 9-month-old mice. This was reflected in a significant increase in the time of latency to target (Figure 1C, *p* < .05) and escape distance to the platform (Figures 1D,E, *p* < .001). In the detection test without the platform, there were fewer that passed the platform area (Figure 1F, *p* < .001).

#### Methanolic extract of the MFS improves cognitive dysfunction in AD mice

**FIGURE 2:**
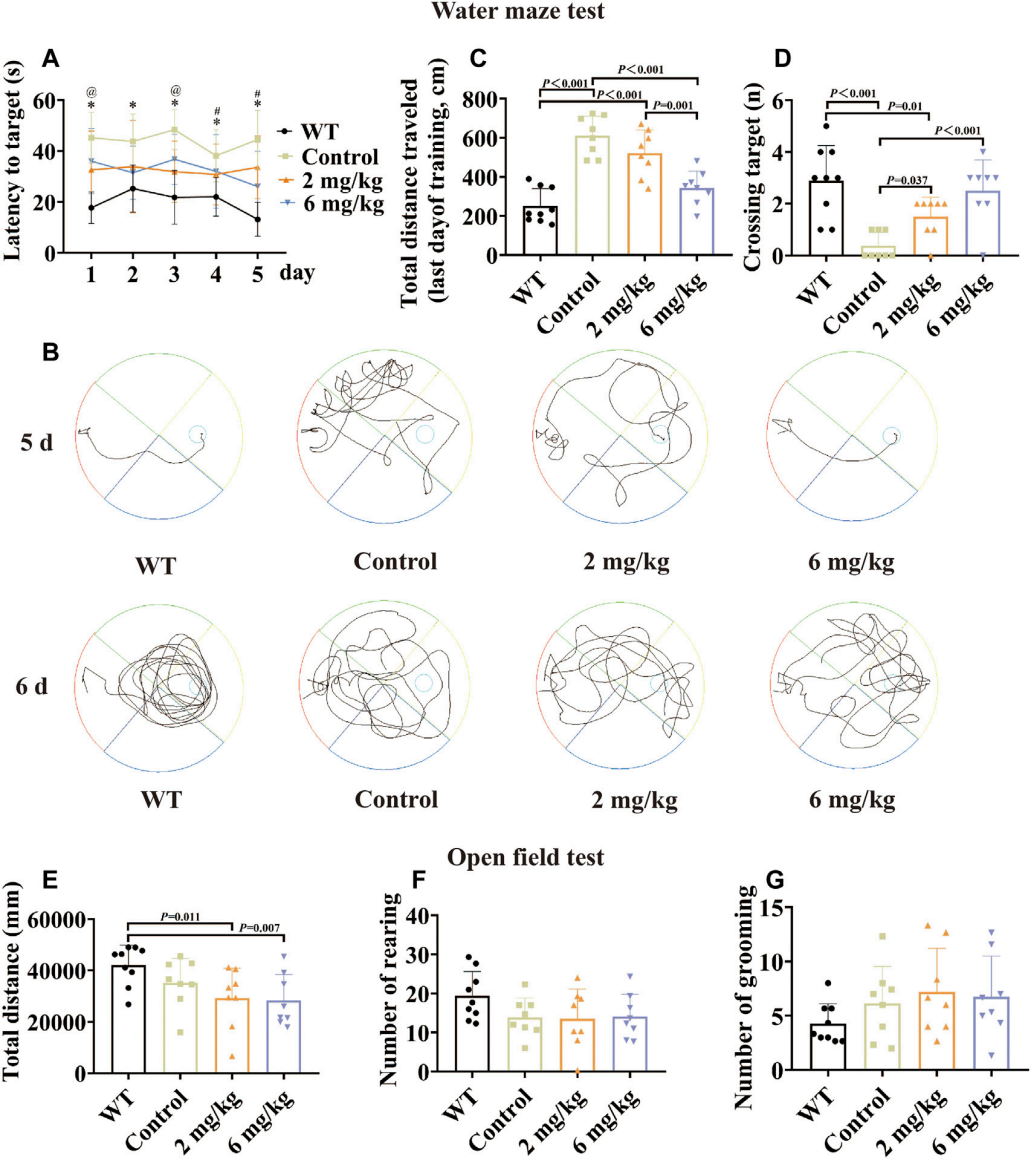
Morris water maze test and open-field test by gavage of the methanol extract of MFS in 10-month-old mice. The Morris water maze test was performed in 10-month-old male WT C57 mice and AD model mice after 1 month of gavage. The results showed that after treatment, the spatial learning and memory functions of AD model mice improved, with the 6 mg/kg group having a more pronounced effect. By 1 month of gavage treatment, in the open-field test, there were no statistical differences in the total distance, number of grooming, and number of rearing in the treated group compared to the control group (data are expressed as mean ± standard deviation, WT, *n* = 9; control, *n* = 8; 2 mg/kg, *n* = 8; 6 mg/kg, *n* = 8). **(A)** Escape latency (seconds) during platform trials. (* indicates WT compared to the control group *p* < .05; @ indicates the control group compared to the 2 mg/kg treatment group *p* < .05; # indicates the control group compared to the 6 mg/kg treatment group *p* < .05). **(B)** Trajectory diagram of the last day of training and day 6 of testing for the Morris water maze test. **(C)** Distance traveled (centimeters) on the last day of training in the Morris water maze test. **(D)** Number of crossings over the target platform of the Morris water maze test at day 6. **(E)** Distance in the open-field test (millimeters). **(F)** Number of rearing. **(G)** Number of grooming.

Compared with the untreated control group, the treated group performed better in the Morris water maze test when given by gavage with the methanol extract of MFSs for 1 month. Among them, the therapeutic effect was more notable in the 6 mg/kg treatment group than in the 2 mg/kg group. This was reflected in a significant reduction in escape latency times and escape to platform distances (Figures 2A,B,C, *p* < .05). A progressive platform learning trial was conducted, and the 6 mg/kg group traversed the platform area more often compared to the control group in the detection trial without a platform (Figure 2D, *p* < .001).

#### Methanolic extract of the MFS has no effect on autonomous and exploratory behaviors in AD mice

To further explore the effects of MFS on mood and activity in mice, an open-field test was conducted on 10-month-old mice. There was no difference in the number of grooming and rearing in the MFS treatment group compared to the control group (Figures 2F,G, *p >* .05), and the total distance (Figure 2E, *p >* .05). In addition, the WT group showed an increase compared to control and treatment groups in total distance (Figure 2E, *p* < .05).

#### Methanolic extract of the MFS treats neuronal damage and neurological deficits

**FIGURE 3:**
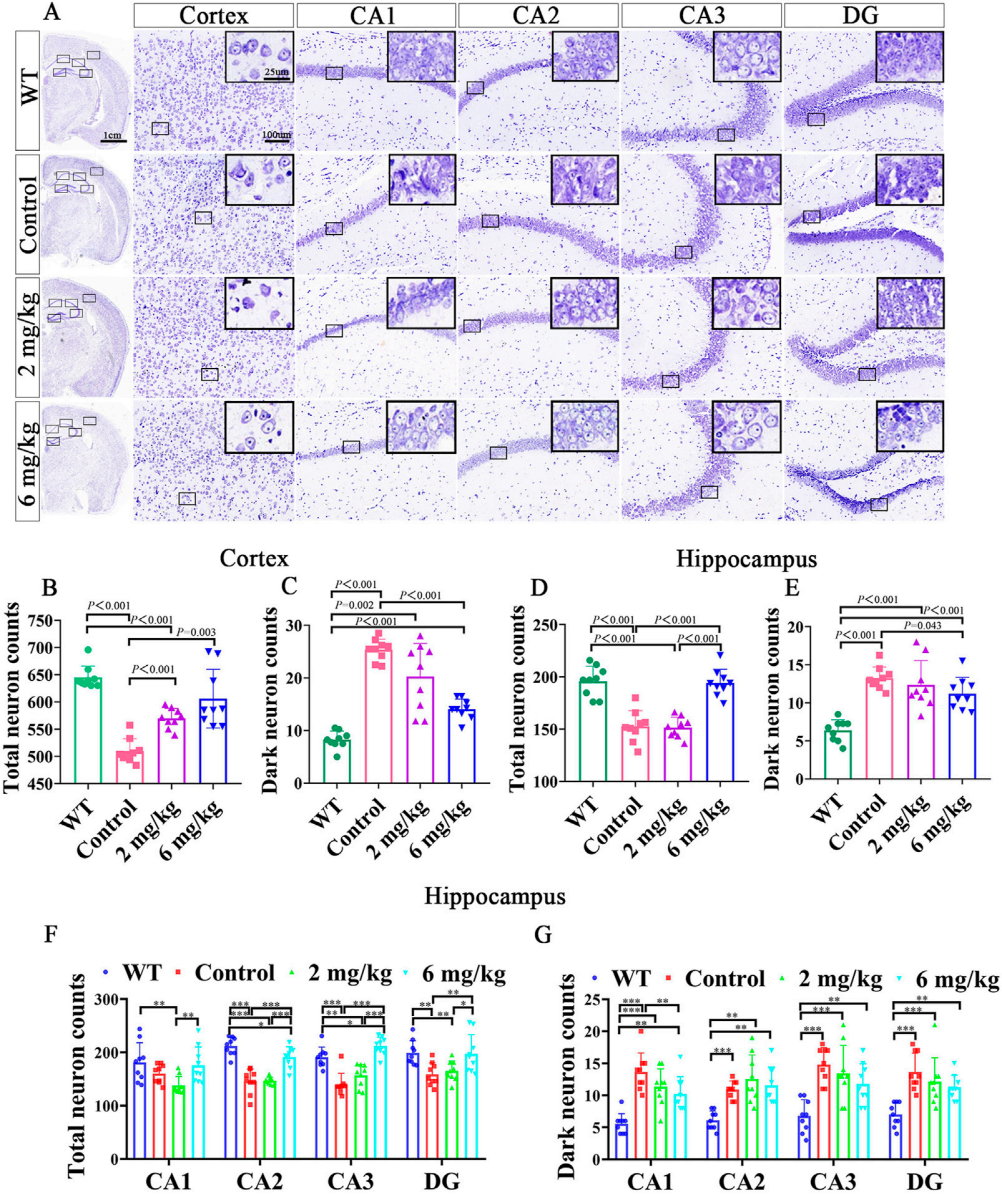
Nissl staining of the brain after gavage treatment for 1 month. After gavage treatment for 1 month, total surviving neurons increased, and dark neurons decreased (DG, dentate gyrus; CA, cornu ammonis) (*n* = 3, each mouse provides three data, data are expressed as mean ± standard deviation). **(A)** Nissl staining of the cortex and hippocampus in WT, control, 2 mg/kg, 6 mg/kg group. **(B)** Quantitative histogram of cortical total surviving neuron statistics in mice. **(C)** Quantitative histogram of cortical dark neuron statistics in mice. **(D)** Quantitative histogram of hippocampal total surviving neuron statistics in mice. **(E)** Quantitative histogram of hippocampal dark neuron statistics in mice. **(F)** Histogram of surviving total neurons in different subdivisions of the mouse hippocampus. **(G)** Histogram of dark neurons in different subdivisions of the mouse hippocampus.

To investigate how the methanolic extract of MFS exhibits its neuroprotective effect on the AD mouse model, Nissl staining was further performed on the mouse brain sections. Also, it was found that the treatment groups treated neuronal damage and neurological deficits compared with the untreated control group of the same age (Figure 3A). Compared with the control group, the treatment group retained more total cortical neurons (6 mg/kg vs. control, *p* = .003; 2 mg/kg vs. control, *p* < .001, Figure 3B) and fewer dark neurons (6 mg/kg vs. control, *p* < .001, Figure 3C). As for the hippocampus, the 6 mg/kg treatment group showed more total neurons retention and fewer dark neurons when compared with the control group (total neurons, 6 mg/kg vs. control, *p* < .001; dark neurons, 6 mg/kg vs. control, *p* = .043), whereas there was no statistical difference in the 2 mg/kg treatment group (Figures 3D,E, *p >* .05).

In the total neuron statistics of different hippocampal subdivisions, the 6 mg/kg group retained more total neurons in CA2, CA3, and DG compared with the control group (*p* < .01). However, there was no statistical difference in the 2 mg/kg treatment group compared with the control group (*p >* .05). Notably, there were fewer total neurons in CA1, CA2, CA3, and DG in the 2 mg/kg treatment group than in the 6 mg/kg group (Figure 3F, *p >* .05). In the different dark neuron regions of the hippocampus, the 6 mg/kg group showed fewer dark neurons in the CA1 region (*p* < .05) than in the control group. In contrast, there was no statistical difference in the 2 mg/kg group compared with the control group and 6 mg/kg treatment group (Figures 3F,G, *p >* .05).

### Acquisition and screening of drug components

**FIGURE 4:**
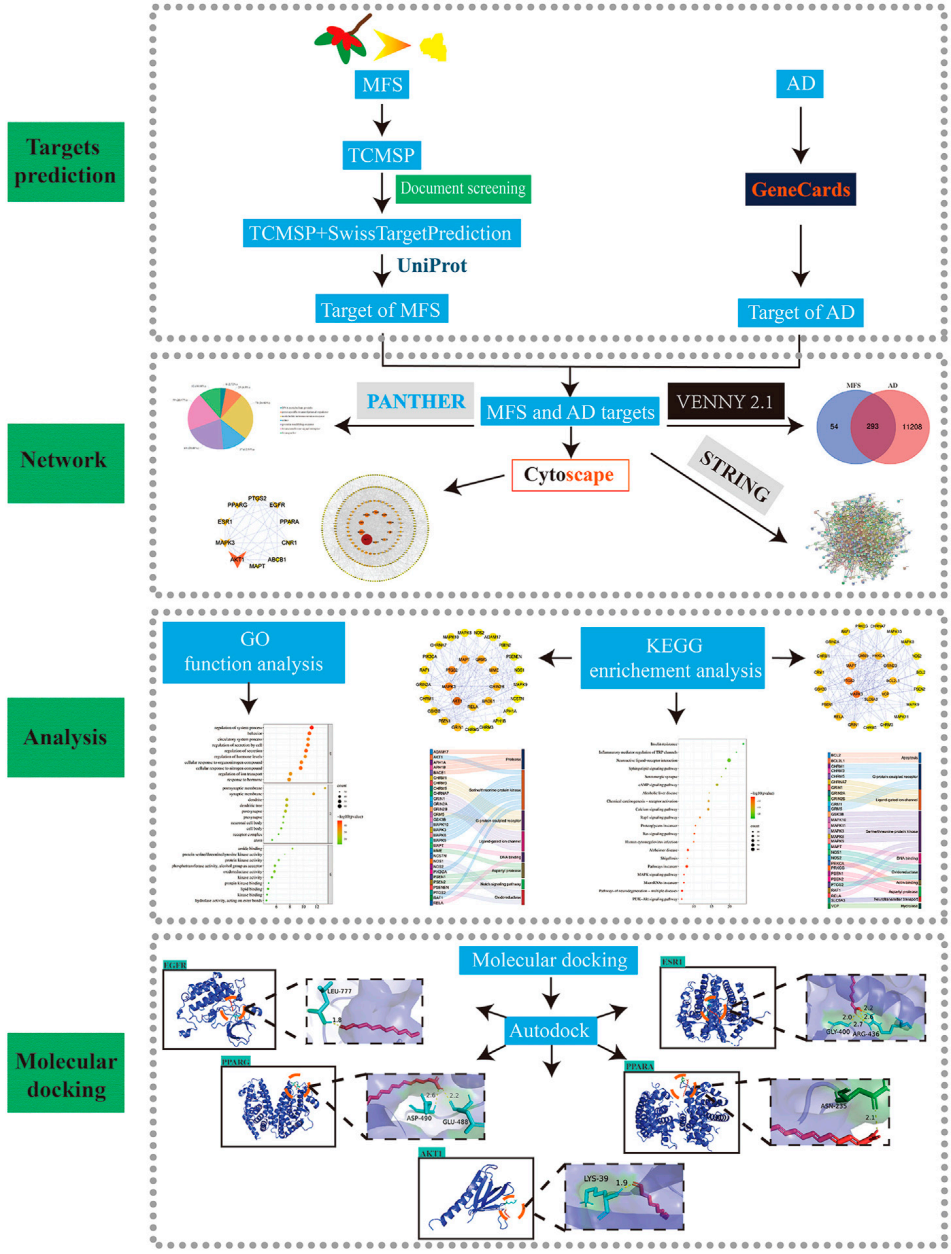
Flow chart of study design. (MFS, miracle fruit seed; AD, Alzheimer’s disease).

To further explore the potential mechanism and key targets of MFS in treating AD, we conducted network pharmacology and molecular docking analysis on drug component action targets and disease pathogenic targets to explore the possible mechanism of MFSs in treating AD (Figure 4) (Wei, et al., 2021). Through the aforementioned research, we found that MFSs have a therapeutic effect on AD. However, the possible mechanisms are still unclear. A total of 74 compounds were found and downloaded through a search of the available open literature (Qi, et al., 2012; Ma, et al., 2016; Jian and Qiao, 2018; Huang et al., 2020). Components with their information are given in Table 3. Of these, chemical components of No. 1–54 can be searched in the TCMSP database. Two components met the requirement: OB ≥ 30% and DL ≥ .18. There were ethyl oleate and 2-monoolein.

**TABLE 3:** Miracle fruit seed screening selected 74 compounds.

No.	Name	MW (g/mol)	OB (%)	DL
1	Palmitic acid	256.48	19.30	.10
2	Stearic acid	284.54	17.83	.14
3	Linoleic	280.50	41.9	.14
4	Myristic acid	228.42	21.18	.07
5	Lauric acid	200.36	23.59	.04
6	Azelex	188.25	16.90	.04
7	Pentadecylic acid	242.45	20.18	.08
8	Methyl undecenate	198.34	40.59	.04
9	Daturic acid	270.51	18.51	.12
10	**Gondoic acid**	310.58	30.7	0.2
11	Octane	114.26	29.72	.01
12	Hexanal	100.18	55.71	.01
13	Cumene	120.21	46.93	.02
14	Valeric acid	102.15	70.74	.01
15	2-Heptenal, (Z)-	112.19	40.19	.01
16	2-Pentylfuran	138.23	54.59	.02
17	Octanal	128.24	19.07	.01
18	Hexanoic acid	116.18	73.08	.01
19	Nonanal	142.27	40.28	.02
20	Naphthalene	128.18	27.55	.03
21	2,4-Decadienal	152.26	51.03	.02
22	2-Undecenal	168.31	39.35	.03
23	Pentadecane	212.47	13.98	.05
24	Methyl 14-methylpentadecanoate	270.51	9.42	.11
25	Oleic acid	282.52	33.13	.14
26	**Ethyl oleate**	310.58	32.40	.19
27	14-Pentadecenoic acid	240.43	36.39	.08
28	**2-Monoolein**	356.61	34.23	.29
29	Lupeol acetate	468.84	9.1	.76
30	Linolenic acid	278.48	45.01	.15
31	Arachic acid	312.60	16.66	.19
32	Eicosenoic acid	310.58	28.64	.20
33	Docosanoate	340.66	15.69	.26
34	Dekan	142.32	17.74	.01
35	2,6-Dimethylheptadecane	268.59	3.81	.1
36	(6R)-2,6-Dimethyloctane	142.32	18.15	.01
37	2-Iodo-2-methylbutane	198.06	9.07	.01
38	Heptadecane	240.53	9.64	.07
39	Undecane	156.35	17.15	.02
40	Octadecane,6-methyl	268.59	10.42	.10
41	4-Ethyl-decane	170.38	6.02	.02
42	3-Methylundecane	170.38	6.57	.02
43	Dodekan	170.38	17.74	.02
44	2,6,10-Trimethyl-dodecane	144.14	37.80	.03
45	Cyclododecane	168.36	48.16	.03
46	3-Methyl-tridecane	198.44	5.24	.04
47	Hexadecane	226.50	12.32	.06
48	Tridecylene	182.39	17.69	.03
49	Pentadecene	210.45	17.72	.05
50	Beta-curcumene	204.39	4.48	.06
51	δ-Cadinol	204.39	17.13	.08
52	(2R)-2-Ethylhexan-1-ol	130.26	26.12	.01
53	Ethol	242.50	13.32	.08
54	Ethylpentadecanoate	270.51	19.74	.11
55	Cis-8-octadecenoic acid	282.5	–	–
56	Cis-10-octadecenoic acid	282.5	–	–
57	9,10-Epoxystearic acid	298.5	–	–
58	2-Dodecenal	182.30	–	–
59	5-Methyltridecane	198.39	–	–
60	5-Ethyl-2,2,3-trimethylheptane	170.33	–	–
61	2,2,4,6,6-Pentamethylheptane	170.33	–	–
62	Ethyl palmitate	284.5	–	–
63	2,2,3,3-Tetramethylpentane	128.25	–	–
64	Tetradecane	198.39	–	–
65	18-Methylnonadecanoic acid	312.5	–	–
66	2-Decyltetradecanoic acid	368.6	–	–
67	Tridecane, 3-methylene	196.37	–	–
68	2,3-Dimethylundecane	184.36	–	–
69	2,8-Dimethylundecane	184.36	–	–
70	2,5-Dimethyldodecane	198.39	–	–
71	2,2,3-Trimethyldecane	184.36	–	–
72	2,2,4,4-Tetramethyloctane	170.33	–	–
73	3,7-Dimethyldecane	170.33	–	–
74	2,3-Dimethyldecane	170.33	–	–

The bold parts are the components that meet the inclusion conditions in the database.

The remaining 20 chemical components are not currently included in the database. Among these 20 compounds, 10 components successfully predicted their targets, including cis-8-octadecenoic acid, cis-10-octadecenoic acid, 9,10-epoxystearic acid, 2-dodecenal, 5-methyltridecane, 5-ethyl-2,2,3-trimethylheptane, 2,2,4,6,6-pentamethylheptane, ethyl palmitate, 2,2,3,3-tetramethylpentane, and tetradecane. The other 10 components were not successfully predicted due to the lack of relevant and similar information in the database, including 18-methylnonadecanoic acid, 2-decyltetradecanoic acid, tridecane, 3-methylene, 2,3-dimethylundecane, 2,8-dimethylundecane, 2,5-dimethyldodecane, 2,2,3-trimethyldecane, 2,2,4,4-tetramethyloctane, 3,7-dimethyldecane, and 2,3-dimethyldecane.

### AD and MFS network construction

**FIGURE 5:**
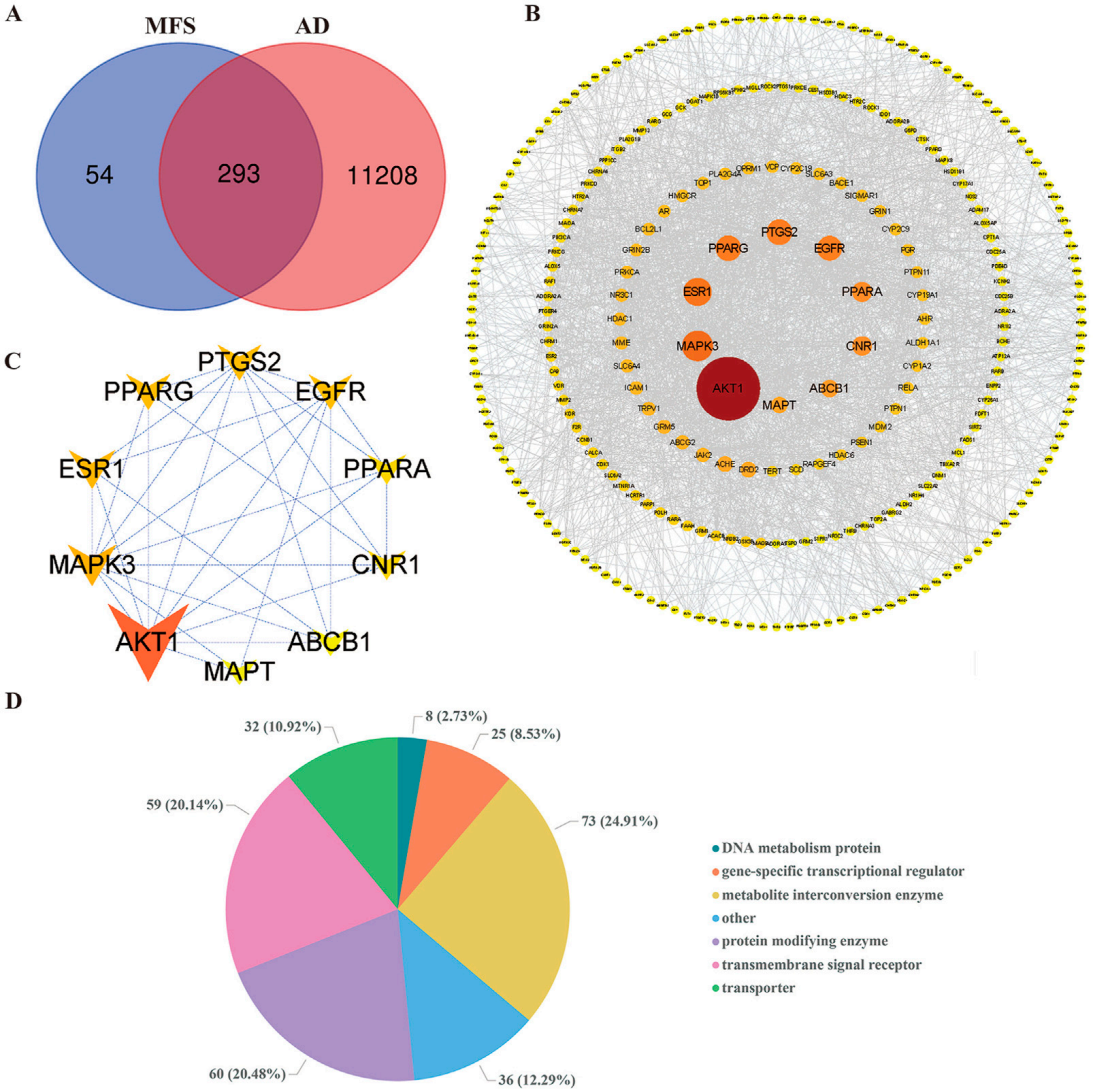
Alzheimer’s disease and miracle fruit-related targets and PPI network. The search obtained 11,501 AD-related targets, 347 MFS-related targets, and 293 disease–drug common targets. The top 10 core targets were screened by betweenness centrality. As the figure shows, the larger area of the circle could be considered as more important in this network. **(A)** Disease–drug target Venn diagram. **(B)** PPI network of drug and disease common targets. **(C)** Core target PPI network. **(D)** Pie chart of the top six molecular functions of MFS-AD common targets.

Among the 12 compounds, 347 targets were obtained from TCMSP and SwissTargetPrediction databases. A total of 111,501 AD-related targets were obtained from the GeneCards database. The intersection of 11,501 AD-related targets and 347 MFS-related targets, and a total of 293 targets were identified as the potential therapeutic targets for MFS in the treatment of AD (Figure 5A). These 293 targets were uploaded to the STRING database, and then the prediction of association between them was performed and then imported into Cytoscape for PPI network construction. In the PPI network, the darker the color of the graph and the larger the area, the higher the BC score is represented. It also means that the target plays a more important role in intercorrelation (Figure 5B). We used the Panther database to perform MF analysis on common targets, and the top six groups include metabolite interconversion enzyme (*n* = 73), protein-modifying enzyme (*n* = 60), transmembrane signal receptor (*n* = 59), transporter (*n* = 32), gene-specific transcriptional regulator (*n* = 25), DNA metabolism protein (*n* = 8), and others (*n* = 36) (Figure 5D). In Figure 5C, the top 10 key genes which ranked by BC scores, were obtained as to be the hub genes, namely, AKT1 (BC = 14,078), MAPK3 (BC = 5,678), ESR1 (BC = 5,017.), PPARG (BC = 4,371), PTGS2 (BC = 4,348), EGFR (BC = 4,198), PPARA (BC = 2,955), CNR1 (BC = 2,856), ABCB1 (BC = 2,291), and MAPT (BC = 2,019).

### GO and KEGG pathway enrichment analyses

**FIGURE 6:**
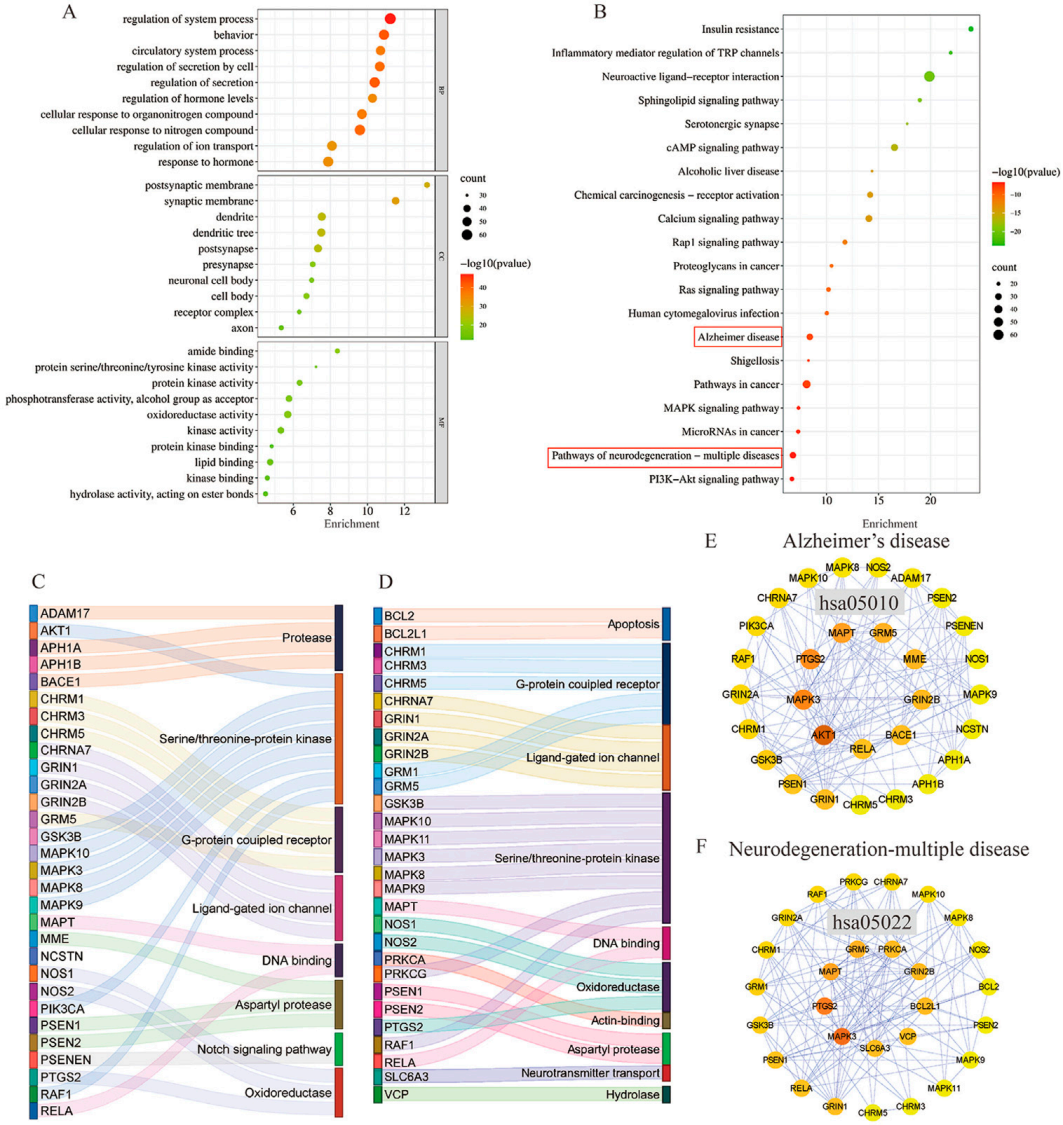
GO function enrichment and KEGG pathway enrichment analysis of AD in the treatment of MFS. **(A)** GO function enrichment analysis bubble diagram. **(B)** KEGG pathway enrichment analysis bubble diagram. **(C)** Sankey diagram of targets involved in the AD signaling pathway. The color of the Sankey diagram indicates the corresponding functional classification of the gene (classification according to MF of each target). **(D)** Sankey diagram of targets involved in the neurodegeneration-multiple disease signaling pathway (classification according to MF of each target). **(E)** PPI network of enriched genes in the AD signaling pathway. **(F)** PPI network of enriched genes in neurodegeneration-multiple disease signaling pathways.

A total of 293 potential therapeutic targets were analyzed using Metascape for GO enrichment analysis. The top 10 significantly enriched terms involving more targets in the BP, MF, and CC, which are shown in Figure 6. MFSs may regulate the system process, behavior, and circulatory system process *via* amide binding, protein kinase activity binding postsynaptic membrane, synaptic membrane, and dendrite to exhibit its therapeutic effect on AD (Figure 6A).

To explore the potential therapeutic mechanism of MFSs in treating AD, 293 common genes were analyzed by pathway enrichment. The top 20 pathways are shown in Figure 6B. Among these potential therapeutic pathways, Alzheimer’s disease, pathways in cancer, and the pathway of neurodegeneration-multiple disease were the most significantly enriched pathways. In addition, insulin resistance, PI3K/Akt signaling pathway, cAMP pathway, and MAPK signaling pathway were also included, which were also important in AD pathology. For further investigation of the mechanistic role of 293 targets in the key pathways, we performed PPI and MFS analyses of the pathways included in the targets. In the Alzheimer’s disease pathway which contained 30 MFS-AD-treating targets, more targets were related to protease and serine/threonine–protein kinase, including two core targets, AKT1 and MAPK3 (Figure 6C). PPI network diagrams were constructed, and AKT1 and MAPK3 were also in key positions (Figure 6E). In the neurodegeneration-multiple disease pathway which contained 29 MFS-AD-treating targets, most targets were associated with serine/threonine–protein kinase and G-protein coupled receptors, including MAPK3 (Figure 6D). The PPI network suggested that MAPK3 may have an important role in MFSs treating AD.

**FIGURE 7:**
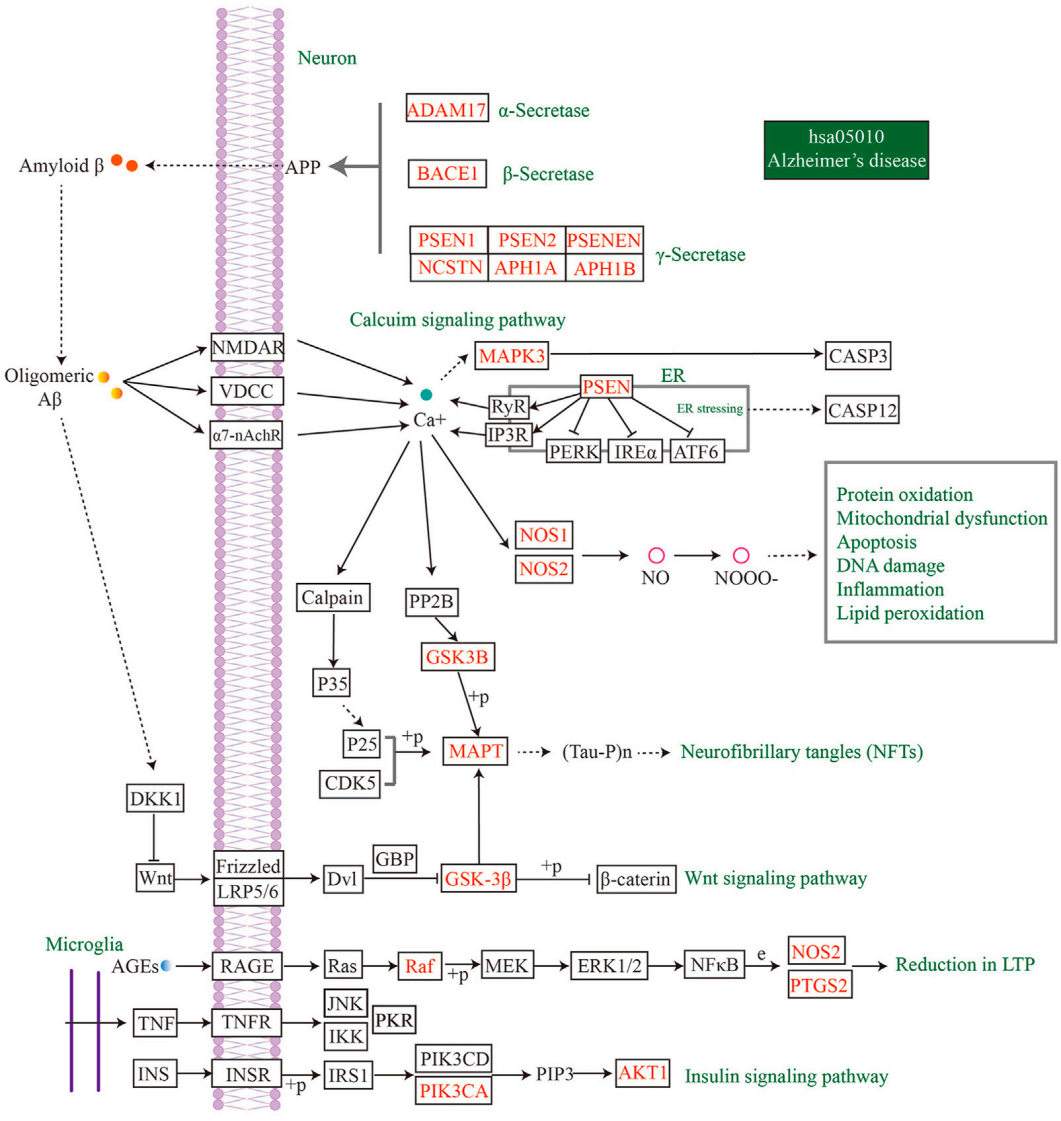
MFS targets involved in the AD pathway (hsa05010) were shown in the mechanism diagram of AD pathology. The target in red font represented the possible therapeutic target of MFS involved in the AD pathway.

To identify the main targets for the treatment of AD in MFS, we visualized the AD pathway in Figure 7. Among them, the red labels represented the genes in the AD pathway among the targets of the MFS effect, which may also be the key targets of the MFS therapeutic AD mechanism. Aβ generation was highly correlated with the enzyme that cleaved APP, including α-secretase (ADAM17), β-secretase (BACE1), and γ-secretase (PSEN1, PSEN2, PSENEN, APH1A, APH1B, and NCSTN). Among Tau-related pathways, the therapeutic effects of MFS may be *via* MAPT, GSK-3β, and GSK3B. Among the MFS-AD targets, AKT1 occupied an important position and was also an essential part of the insulin resistance-related AD disease pathway.

### Molecular docking analysis of MFS-AD targets

**FIGURE 8:**
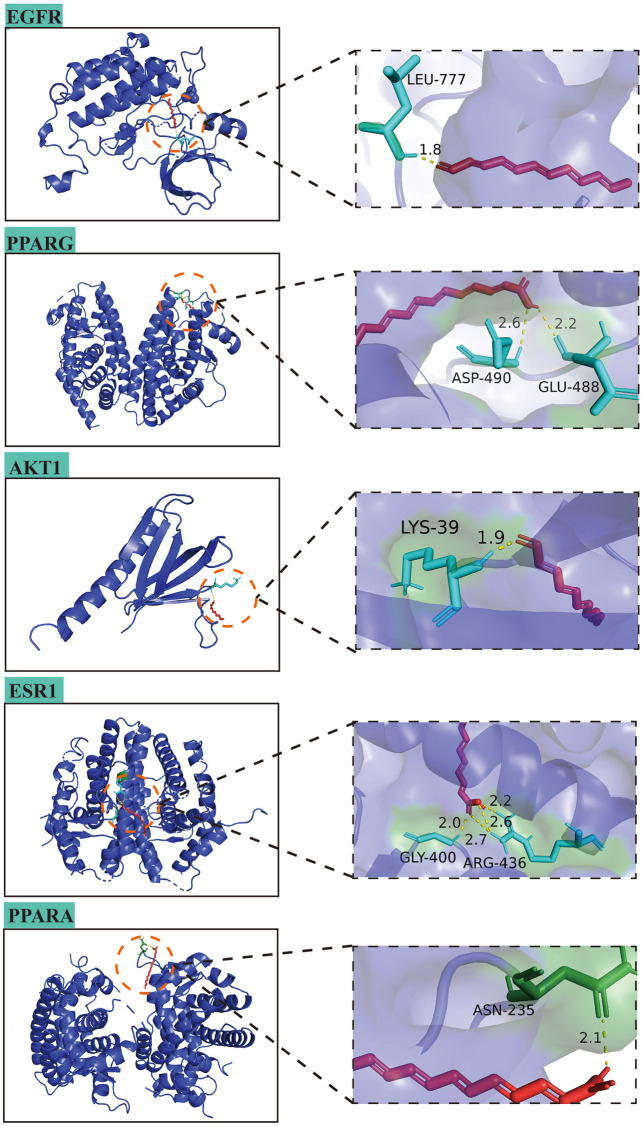
Molecular docking diagrams of AD-MFS and top five hub genes.

In this study, the interaction of 10 core targets and MFS was verified by molecular docking. In the process of molecular docking, target genes were applied, AKT1 (PDB:1UNP), MAPK3 (PDB:6GES), ESR1 (PDB:6PSJ), PPARG (PDB:8DSZ), PTGS2 (PDB:5F19), EGFR (PDB:5HG8), PPARA (PDB:3ET1), CNR1 (PDB:5U09), ABCB1 (PDB:7A69), and MAPT (PDB:4E0N). Figure 8 shows the top five highest binding energies of MFSs to key amino acids for demonstration. The results of the binding free energy indicate that the core components of MFS have good binding activity to the core target, which were −6.04, −4.63, −4.43, −4.31, and −4.05 kcal Mol ^−1^ (Table 4).

**TABLE 4:** Docking results of target protein and binding energy.

Core target	PDB ID	Binding energy/(kcal Mol ^−1^)
EGFR	5HG8	−6.04
PPARG	8DSZ	−4.63
AKT1	1UNP	−4.43
ESR1	6PSJ	−4.31
PPARA	3ET1	−4.05

## Discussion

Currently, the number of people with AD is increasing every year. However, due to the complex mechanisms, multiple protein, and pathways involved in the development and progression of AD, there is no definitive treatment. In this study, we first discovered that MFSs have a therapeutic effect on AD and revealed the possible mechanism from network pharmacology and molecular docking, which provides a new idea for the treatment of AD. First, we performed behavioral screening of the AD mouse model (APP/PS1 overexpression) and found significant cognitive dysfunction at nine months of age, similar to the behavioral experiments observed in the literature (Yao, et al., 2015). Then, we treated the AD mouse model with the MFS methanolic extract for 1 month and found that the learning and memory function was improved. Finally, to explore the possible mechanisms of MFS for AD, we performed an analysis using network pharmacology and molecular docking and found an important role of AKT1 and the insulin pathway in it.

At present, plant therapy for AD has attracted more and more attention because of its comprehensive effects and less side effects (Mithu et al., 2014; Sun et al., 2014; Yan et al., 2017; Martínez-Coria et al., 2019; Singh et al., 2019; Baranowska-Wójcik et al., 2020; Hosseini et al., 2021; Nowak et al., 2021; Sohn et al., 2021; Anupama et al., 2022). The mystery fruit, which we used in this study, has also received more attention due to its good antioxidant properties, including lowering cholesterol (Huang et al., 2020), antidiabetic effects (Obafemi, et al., 2019), reducing serum uric acid levels (Shi et al., 2016), and anti-oxidative damage (Chen et al., 2015). In addition, 12 phenolic substances were identified and quantified in the flesh of the mystery fruit, demonstrating that the mystery fruit is an antioxidant-rich fruit with an important role in scavenging free radicals (Du, et al., 2014). Polyphenols have been shown to have great potential in the treatment of degenerative diseases, especially in AD (Sylla, et al., 2015; Nabavi, et al., 2018; Seo, et al., 2018; Reddy, et al., 2020). MFSs have been reported to act on the insulin pathway, increasing insulin synthesis and reducing inflammation, which was an important reason that motivated us to use it as a potential therapeutic approach for AD (Ma, et al., 2016; Obafemi, et al., 2019).

In the experiment, we clarified the therapeutic significance of MFSs on AD memory function using the Morris water maze test. Regarding the research on AD, it has also been recently found that memory disorders are accompanied by mood disorders, including anxiety and depression, in the early stages (Mendez, 2021; Pentkowski, et al., 2021). As previously reported, in the APP/PS1 model mice, we also have observed an increase in anxiety-like behavior (Guo, et al., 2012). The open-field test is a common method for assessing anxiety-like behavior and has now been used in AD transgenic rodents to detect anxiety. This was manifested by a decrease in total distance and the number of rearing, and an increase in the number of grooming in the open-field test. However, after one month of treatment with MFSs, the anxiety behavior of AD model mice was not improved. This implies that the short-term treatment of MFSs has an efficacy on memory improvement in AD, but no effect on anxiety. This may require longer term treatment to demonstrate whether there is a clear effect on mood.

This study identified a therapeutic effect of MFSs for AD, but the mechanism involved is unclear. We found more surviving neurons in cortical and hippocampal regions in the treatment group through Nissl staining, especially in the 6 mg/kg treatment group. In the case of AD, learning and memory dysfunction is one of the critical manifestations. The cerebral cortex and hippocampus are closely related to the cognitive function of the brain (Hu, et al., 2019). Atrophy of the medial temporal lobe and hippocampal region was also found during the progression of AD (Ferreira, et al., 2020). The hippocampus is one of the important brain regions involved in learning and memory regulation affected by AD and belongs to the limbic lobe, which is subdivided into the DG and parts of CA. In the DG region of the adult hippocampus, there are still newborn hippocampal neurons. Newborn neurons in the DG region not only project to CA2 but also promote excitation of CA3 vertebral neurons, affecting memory formation and recording (Llorens-Martín, et al., 2015). The proliferation of such neurons is an important link in the preservation of hippocampal function, including memory, learning, and emotion (Rao, et al., 2022). Previous studies have shown significant neuronal loss in the CA1 and DG regions of the hippocampus at 16 months of age in APP/PS1 mice (Chao, et al., 2018). In the current research, it was found that neuronal loss was observed in both APP/PS1 (Ou, et al., 2018) and AD patients (Stygelbout, et al., 2014). Unlike most areas of the adult mammalian brain, neural stem cells with the capacity to generate new neurons are present in the hippocampus, a process known as neurogenesis (Vieira, et al., 2018). Hippocampal neural stem cells are found mainly in the subgranular zone and constitute most excitatory neurons in the DG (Babcock, et al., 2021). Several studies have shown that adult hippocampal neurogenesis produces neurons that are important for learning memory and emotion regulation (Sahay, et al., 2011; Anacker and Hen, 2017; Sakalem, et al., 2017). In our study, we found that MFSs significantly increased the number of neurons and inhibited the apoptosis of hippocampal neurons, especially in the DG. These results suggest that MFSs not only repaired damaged neurons but also prevented the loss of neurons in the hippocampal region of AD mice, which may be related to neurogenesis in the DG region. It might explain the improvement of learning and memory we observed in MFS-treated AD mice.

As with many natural plants to a point, MFS affects many targets in the treatment of AD. Based on the literature and database information, we have described the relatively important target. AKT is a serine/threonine protein kinase, which is activated by the insulin signal to promote cell survival, cell growth, cell proliferation, and regulate glucose/lipid metabolism (Manning and Cantley, 2007). Insulin signaling is genetically stable as an evolutionarily conserved pathway. Insulin is a protein hormone secreted by the β cells of the pancreas, stimulated by endogenous or exogenous substances such as glucose, lactose, ribose, arginine, and glucagon. It is also the only hormone *in vivo* that lowers blood glucose and promotes glycogen, fat, and protein synthesis. Regarding AD, more therapeutic mechanisms are also being investigated, and one of the areas is impaired brain metabolism, especially the role of insulin. Insulin has long been thought to be associated with AD (Akhtar and Sah, 2020; Spinelli, et al., 2020). Insulin has a role in promoting dendritic spine formation and nutritional synapses in the brain (Lee, et al., 2011). In some studies, treatment with insulin and medications that promote insulin signaling have also been found to improve neuropathology and as cognition in AD with diabetes (Craft, et al., 2017; Kellar, et al., 2021). PI3K/Akt signaling pathways are also considered to be classical pathways affecting insulin secretion. A study showed that insulin resistance was positively correlated with Aβ deposition in the frontal and temporal regions of the brain in insulin-resistant but normoglycemic AD patients (Willette, et al., 2015). This indicates that the onset of insulin resistance may precede the deposition of Aβ in the pathology process of AD. Notably, it was found that defective insulin signaling in the brain of AD patients may contribute to synaptic damage and cognitive deficits, and that normalization of insulin signaling may be beneficial (Gralle, 2017; Ferrario and Reagan, 2018). Abnormal insulin signaling leads to impairment of the PI3K/Akt signaling pathway, causing oxidative stress, impaired energy metabolism, neuroinflammation, mitochondrial dysfunction, autophagy dysfunction, and neurogenic death, all of which promote the development of AD and exacerbate cognitive dysfunction. AKT1 has been implied to be involved in insulin signaling, associated with the AD pathological process, so AKT1 may be a potential mechanism for MFS treatment of AD.

To further investigate the effect of MFS on AD, we used molecular docking to study the binding ability of MFS to the target protein. The results showed that MFS binds most stably to EGFR with a binding energy of −6.04 kcal Mol ^-1^. EGFR is a transmembrane protein and pro-activation of EGFR is one of the most common pathogenic driver events under various inflammatory conditions, including neurodegenerative diseases such as AD (Tavassoly and Tavassoly, 2021). In the embryonic brain, EGFR expression is essential for strengthening neural axon growth (Lu, et al., 2014). In the adult brain, EGFR expression may be elevated again by the presence of inflammation, especially in the subventricular zone, hippocampus, and cerebellum (Romano and Bucci, 2020). Hyperphosphorylation of EGFR in AD activates GSK-3 and eventually dephosphorylates Akt, leading to reduced β-linked protein signaling and Wnt signaling pathways (Krejci, et al., 2012). This leads to abnormal neuronal energy metabolism, cytoskeletal dysregulation, and reduced autophagy (Jayaswamy, et al., 2022). These factors affect synapses and lead to amyloidogenic pathway activation (Palomer, et al., 2019). Recent studies on AD using EGFR inhibitors were also found to improve memory function in APP/PS1 mice (Jayaswamy et al., 2022) and to mediate autophagy in early AD (Wang, et al., 2017). Therefore, EGFR inhibition may be a potential modality for AD treatment and may be one of the mechanisms of action of MFS for AD.

Among the common targets of AD and MFS, most of them were enriched in the AD pathway, including the insulin signaling pathway, Wnt signaling pathway, and calcium signaling pathway (Figure 7). Also, AKT1 and EGFR have important roles in these pathways. Therefore, MFSs may play a therapeutic role in AD by affecting the insulin pathway and Wnt signaling pathway through genes such as AKT and EGFR. This study is a preliminary effect analysis and will be followed by the isolation of MFS monomer for the treatment of AD and the search for natural anti-AD ingredients.

## Conclusion

In conclusion, our study suggests that MFSs could be a potential treatment for AD. Moreover, this therapeutic mechanism may be achieved by increasing surviving neurons and affecting the insulin pathway and Wnt pathway signaling pathways. In addition, this discovery not only expands the medicinal value of MFS, but also opens up more possibilities for phytological treatment options for AD. Further studies should be conducted to identify the components behind the medicinal properties of MFS and the corresponding mechanisms of action.

## Limitation

Of course, there are also some limitations in this study. First, there is a lack of further research on drugs, including *in vivo* metabolism and toxicology. The drug composition lacks *in vivo* data support, and the experimental group will follow up with experiments on drug monomers for the treatment of AD. Second, the mechanism of MFS in treating AD lacks further molecular mechanism research and *in vitro* research. Third, the current network pharmacology is a static point analysis, lacking more powerful research on the dynamic changes of diseases and chemical changes in the internal process.

## Data Availability

The datasets presented in this study can be found in online repositories. The names of the repository/repositories and accession number(s) can be found in the article/Supplementary Material.
